# Association between life’s essential 8 and hyperuricemia among adults in the United States: insights from NHANES 2005–2018

**DOI:** 10.3389/fmed.2024.1455164

**Published:** 2024-11-06

**Authors:** Xiaolan Wang, Jingxiu Fan

**Affiliations:** ^1^Department of Critical Care Medicine, West China Hospital, Sichuan University, Chengdu, China; ^2^West China School of Nursing, Sichuan University, Chengdu, China; ^3^Department of Cardiovascular Surgery, West China School of Medicine, West China Hospital, Sichuan University, Chengdu, Sichuan Province, China

**Keywords:** hyperuricemia, life’s essential 8, cardiovascular health, NHANES (National Health and nutrition examination survey), cross-sectional study

## Abstract

**Background:**

Hyperuricemia is a significant risk factor for various metabolic and cardiovascular conditions. Life’s Essential 8 (LE8), a comprehensive measure of cardiovascular health promoted by the American Heart Association, may have a protective role against hyperuricemia. This study aims to evaluate the association between LE8 scores and hyperuricemia in a representative sample of US adults.

**Methods:**

We conducted a cross-sectional study using data from the National Health and Nutrition Examination Survey (NHANES) 2005–2018, encompassing 26,885 adults. LE8 scores were calculated based on diet, physical activity, nicotine exposure, sleep health, body mass index, blood lipids, blood glucose, and blood pressure. Hyperuricemia was defined as serum uric acid levels ≥7.0 mg/dL in men and ≥ 6.0 mg/dL in women. Logistic regression and generalized additive models (GAMs) were used to analyze the relationship between LE8 scores and hyperuricemia, adjusting for potential confounders.

**Results:**

Higher LE8 scores were significantly associated with lower odds of hyperuricemia (OR per 10-point increase: 0.73, 95% CI: 0.72–0.75, *p* < 0.001). Stratified analyses revealed consistent protective effects across subgroups defined by sex, age, race/ethnicity, PIR (poverty income ratio), education level, drinking status, eGFR, and CVD status. Logistic regression and GAM analyses both confirmed a linear relationship between increasing LE8 scores and reduced hyperuricemia risk. For example, in males, the OR was 0.81 (95% CI: 0.78–0.84), and in females, it was 0.66 (95% CI: 0.64–0.68).

**Conclusion:**

The findings suggest that higher LE8 scores are robustly associated with lower odds of hyperuricemia in US adults. These results support the promotion of comprehensive cardiovascular health behaviors encapsulated by LE8 to mitigate hyperuricemia risk. Further studies are needed to explore the causal pathways and potential interventions.

## 1 Introduction

Hyperuricemia, characterized by elevated serum uric acid levels, is a significant precursor to gout and is associated with several metabolic and cardiovascular diseases (CVDs) (1). The prevalence of hyperuricemia has been rising globally (2, 3), correlating with increased rates of obesity, hypertension, and other lifestyle-related conditions (4, 5). Given its multifactorial etiology, addressing hyperuricemia requires a comprehensive approach that includes lifestyle modification and management of underlying risk factors.

Recently, the American Heart Association introduced Life’s Essential 8 (LE8), an updated framework for cardiovascular health that emphasizes a holistic approach to health behaviors and factors (6). LE8 includes eight critical components: diet, physical activity, nicotine exposure, sleep health, body mass index (BMI), blood lipids, blood glucose, and blood pressure. Each of these elements has been individually associated with hyperuricemia and related metabolic disorders (7). For instance, poor diet and physical inactivity contribute to obesity, a major risk factor for hyperuricemia (8). Similarly, smoking and inadequate sleep have been linked to higher serum uric acid levels and increased risk of gout (9, 10). By integrating these components, LE8 offers a comprehensive framework for evaluating and improving overall health, which could potentially mitigate the risk of hyperuricemia.

Previous studies have shown an inverse relationship between LE8 and hyperuricemia, but there are still several limitations. Research conducted in non-U.S. populations has limited generalizability, while studies using U.S. data often lack comprehensive adjustments for key confounders such as socioeconomic status, alcohol consumption, kidney function, and CVD (11–13). Moreover, while some studies have used advanced models to explore non-linear trends, they did not assess the individual contributions of LE8 components to hyperuricemia risk (14). This study addresses these gaps by using a large, representative U.S. dataset, adjusting for a wide range of confounders, and analyzing both overall LE8 scores and individual components, offering a more detailed and precise understanding of LE8’s impact on hyperuricemia.

This study seeks to address this gap by utilizing the National Health and Nutrition Examination Survey (NHANES) data to evaluate the relationship between LE8 scores and hyperuricemia in U.S. adults. Specifically, we assess the combined effects of all LE8 components, offering new insights into the link between comprehensive cardiovascular health and metabolic disorders.

## 2 Materials and methods

### 2.1 Study design and participants

The data from the NHANES 2005–2018 were utilized in this cross-sectional study. NHANES is a program of studies designed to assess the health and nutritional status of adults and children in the United States. It combines interviews and physical examinations to provide a comprehensive dataset. For this analysis, we included adults aged 18 years and older with complete data on serum uric acid levels and LE8 components. Participants with missing data on key variables were excluded, resulting in a final analytical sample of 26,885 individuals.

### 2.2 Demographic characteristics

The demographic characteristics collected in this study included a wide array of variables such as age, sex, race/ethnicity, poverty income ratio (PIR), and education level. Lifestyle factors were also assessed, including smoking status and alcohol consumption. Physical examination data covered measurements of height, waist circumference (WC), blood pressure, and BMI, which was calculated from height and weight. Laboratory data included serum uric acid levels and estimated glomerular filtration rate (eGFR). Additionally, self-reported health information related to CVD history was recorded, encompassing conditions like heart attack, congestive heart failure, coronary heart disease, angina, and stroke. The PIR was calculated by dividing the household income by the poverty threshold, classifying participants into three income groups: low (less than 1.3), medium (1.3 to 3.5), and high (greater than 3.5). Alcohol intake was categorized into five groups: current drinking groups include heavy (defined as consuming 3 or more drinks per day for females, 4 or more drinks per day for males, or engaging in binge drinking on 5 or more days per month), moderate (2 or more drinks per day for females, 3 or more drinks per day for males, or binge drinking on 2 or more days per month), and mild (up to 1 drink per day for females and up to 2 drinks per day for males). Additionally, individuals classified as never drinkers are those who have had less than 12 drinks in their lifetime, while former drinkers are defined as individuals who had 12 or more drinks in 1 year but did not drink in the previous year, or those who did not drink in the previous year but had 12 or more drinks in their lifetime. The eGFR was calculated using the 2009 Chronic Kidney Disease Epidemiology Collaboration (CKD-EPI) formula based on serum creatinine levels (15).

### 2.3 Measurement of LE8

LE8, as defined by the American Heart Association, includes eight components: diet, physical activity, nicotine exposure, sleep health, BMI, blood lipids, blood glucose, and blood pressure (6). Each component is assigned a score based on predefined values, ranging from 0 to 100, with higher scores indicating better health status (Supplementary Table S1). The overall LE8 score is calculated as the arithmetic mean of the scores for these eight components. Based on the overall score, LE8 scores are categorized into three levels: low (below 50), moderate (ranging from 50 to 79), and high (80 or above) (6).

Dietary intake was assessed using the Healthy Eating Index 2015 (HEI-2015), which evaluates diet quality based on key food and nutrient components (Supplementary Table S2) (16). Physical activity was measured in minutes per week of moderate to vigorous activity. Nicotine exposure was determined based on self-reported smoking status and exposure to secondhand smoke. Sleep health was assessed by self-reported average hours of sleep per night. BMI was calculated as weight in kilograms divided by height in meters squared. Blood lipids were measured using non-high-density lipoprotein (HDL) cholesterol. Blood glucose levels were assessed using fasting blood glucose or hemoglobin A1c (HbA1c). Blood pressure was measured using standard procedures during the physical examination.

### 2.4 Measurements and definition of hyperuricemia

Hyperuricemia was defined as serum uric acid levels ≥7.0 mg/dL for men and ≥ 6.0 mg/dL for women, consistent with established clinical thresholds (17). Serum uric acid was measured using a biochemical analyzer with enzymatic methods. Blood samples were collected and processed following standardized NHANES protocols to ensure accuracy and reliability.

### 2.5 Statistical analysis

Descriptive statistics were used to summarize baseline characteristics of the study population. Continuous variables were reported as means and standard deviations, while categorical variables were presented as frequencies and percentages. Logistic regression models were employed to examine the association between LE8 scores and hyperuricemia, adjusting for potential confounders. Three models were constructed: Model 1 was unadjusted, Model 2 was adjusted for age and sex, and Model 3 was further adjusted for race/ethnicity, PIR, education level, alcohol consumption, eGFR, and CVD. We utilized generalized additive models (GAMs) to explore potential non-linear relationships between LE8 scores and hyperuricemia. GAMs offer greater flexibility than traditional linear regression models by allowing for smooth, non-parametric functions of predictor variables. This flexibility helps reduce the risk of model misspecification, particularly when the relationship between predictors and outcomes is complex or unknown (18). GAMs were fitted using the mgcv package in R, with smoothing splines for LE8 scores to capture the trend across its entire range. We selected the degree of smoothness using restricted maximum likelihood (REML) to avoid overfitting. Stratified analyses were conducted to verify the consistency of associations across subgroups defined by sex, age, race/ethnicity, PIR, education level, drinking status, eGFR, and CVD status. Missing data were handled using Multiple Imputation by Chained Equations (MICE) to reduce potential bias and improve estimate precision (19, 20). We generated 5 imputed datasets (m = 5) with 50 iterations (maxit = 50) to ensure stable imputation. A random seed (seed = 500) was set to allow reproducibility. The imputation process was performed using the mice() function, which assesses convergence implicitly by monitoring parameter stability across iterations. A maximum of 50 iterations (maxit = 50) was allowed to ensure convergence. A *p*-value of less than 0.05 was considered statistically significant. Statistical analyses were conducted utilizing R software (version 4.2.0) and EmpowerStats.^1^

## 3 Results

### 3.1 Participant selection and categorization

**Figure 1:**
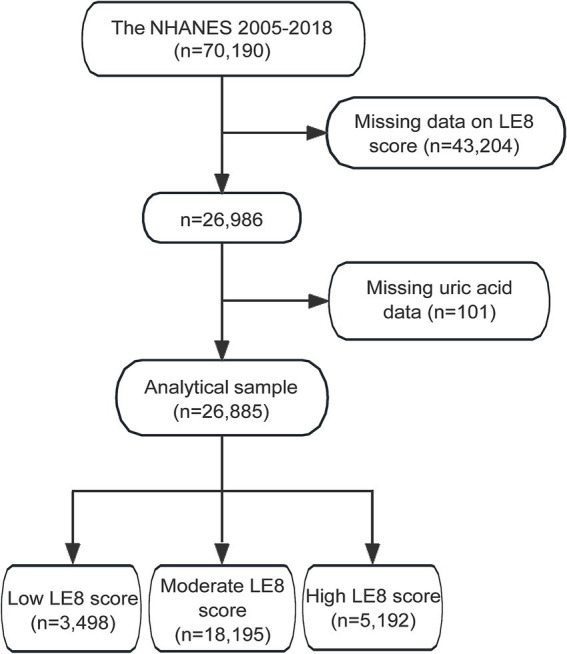
Flowchart of study participants selection from NHANES 2005–2018. NHANES, the National Health and Nutrition Examination Survey; LE8, Life’s Essential 8.

The initial dataset from NHANES 2005–2018 included 70,190 participants. After excluding 43,204 participants with missing LE8 score data, the sample size was reduced to 26,986 participants. Further exclusion of 101 participants with missing uric acid data resulted in an analytical sample of 26,885 participants. These participants were categorized based on their LE8 scores into three groups: 3,498 participants with a low LE8 score, 18,195 participants with a moderate LE8 score, and 5,192 participants with a high LE8 score (Figure 1).

### 3.2 Baseline demographic characteristics

The study population comprised 26,885 participants from the NHANES 2005–2018 dataset, divided into 21,465 individuals without hyperuricemia and 5,420 with hyperuricemia. Significant differences were observed between these groups. Participants with hyperuricemia were older, with a mean age of 54.28 years compared to 48.87 years in the non-hyperuricemia group (*p* < 0.001). Gender distribution also showed a higher proportion of males in the hyperuricemia group (54.74%) than in the non-hyperuricemia group (47.23%), indicating a significant difference (*p* < 0.001). Racial disparities were evident, with a higher percentage of Non-Hispanic Black people in the hyperuricemia group (24.54% vs. 19.53%) and fewer Mexican Americans (10.74% vs. 16.18%) compared to the non-hyperuricemia group (*p* < 0.001). PIR categories revealed that a larger proportion of hyperuricemia participants were in the medium PIR category (39.93%) compared to the non-hyperuricemia group (37.83%; *p* = 0.015). Additionally, education levels showed a higher percentage of high school graduates among the hyperuricemia group (24.50% vs. 22.69%; *p* = 0.018). Drinking habits also differed significantly, with more former drinkers in the hyperuricemia group (*p* < 0.001).

Anthropometric and health measures further highlighted the differences between the groups. Participants with hyperuricemia had higher mean BMI (32.42 kg/m^2^ vs. 28.37 kg/m^2^), WC (108.12 cm vs. 97.37 cm), systolic blood pressure (SBP; 127.80 mmHg vs. 122.55 mmHg), and diastolic blood pressure (DBP; 71.03 mmHg vs. 70.24 mmHg; *p* < 0.001 for all). The eGFR was significantly lower in the hyperuricemia group (81.25 mL/min/1.73 m^2^ vs. 96.12 mL/min/1.73 m^2^; *p* < 0.001). Furthermore, the prevalence of CVD was markedly higher among those with hyperuricemia (17.79% vs. 9.38%; *p* < 0.001; Table 1).

**Table 1:** Baseline characteristics of the study population.

Characteristics	Total *n* = 26,885	Non-hyperuricemia *n* = 21,465	Hyperuricemia *n* = 5,420	*p*-value
Age (years)	49.96 ± 17.58	48.87 ± 17.36	54.28 ± 17.77	<0.001
Sex (n, %)				<0.001
Male	13,105 (48.74%)	10,138 (47.23%)	2,967 (54.74%)	
Female	13,780 (51.26%)	11,327 (52.77%)	2,453 (45.26%)	
Race/ethnicity (n, %)				<0.001
Non-Hispanic White	12,150 (45.19%)	9,548 (44.48%)	2,602 (48.01%)	
Non-Hispanic Black	5,522 (20.54%)	4,192 (19.53%)	1,330 (24.54%)	
Mexican American	4,055 (15.08%)	3,473 (16.18%)	582 (10.74%)	
Others	5,158 (19.19%)	4,252 (19.81%)	906 (16.72%)	
PIR (n, %)				0.015
Low	8,061 (29.98%)	6,465 (30.12%)	1,596 (29.45%)	
Medium	10,284 (38.25%)	8,120 (37.83%)	2,164 (39.93%)	
High	8,540 (31.76%)	6,880 (32.05%)	1,660 (30.63%)	
Education level (n, %)				0.018
Less than high school	6,074 (22.59%)	4,865 (22.66%)	1,209 (22.31%)	
High school	6,199 (23.06%)	4,871 (22.69%)	1,328 (24.50%)	
More than high school	14,612 (54.35%)	11,729 (54.64%)	2,883 (53.19%)	
Drinking (n, %)				<0.001
Never	3,704 (13.78%)	3,011 (14.03%)	693 (12.79%)	
Former	4,486 (16.69%)	3,471 (16.17%)	1,015 (18.73%)	
Mild	9,109 (33.88%)	7,327 (34.13%)	1782 (32.88%)	
Moderate	4,307 (16.02%)	3,478 (16.20%)	829 (15.30%)	
Heavy	5,279 (19.64%)	4,178 (19.46%)	1,101 (20.31%)	
Smoking (n, %)				<0.001
Never	14,843 (55.21%)	12,066 (56.21%)	2,777 (51.24%)	
Former	6,729 (25.03%)	5,031 (23.44%)	1,698 (31.33%)	
Now	5,313 (19.76%)	4,368 (20.35%)	945 (17.44%)	
Height (cm)	167.32 ± 10.11	167.11 ± 10.04	168.16 ± 10.31	<0.001
BMI (kg/m^2^)	29.19 ± 6.84	28.37 ± 6.37	32.42 ± 7.61	<0.001
WC (cm)	99.54 ± 16.37	97.37 ± 15.58	108.12 ± 16.60	<0.001
SBP (mmHg)	123.61 ± 18.30	122.55 ± 17.90	127.80 ± 19.23	<0.001
DBP (mmHg)	70.40 ± 11.81	70.24 ± 11.47	71.03 ± 13.02	<0.001
eGFR (mL/min/1.73 m^2^)	93.12 ± 23.61	96.12 ± 21.93	81.25 ± 26.13	<0.001
CVD (n, %)	2,977 (11.07%)	2013 (9.38%)	964 (17.79%)	<0.001
Uric acid (mg/dL)	5.46 ± 1.43	4.95 ± 1.02	7.46 ± 1.02	<0.001
LE8	66.45 ± 14.47	67.91 ± 14.34	60.69 ± 13.53	<0.001

PIR, poverty income ratio; BMI, body mass index; WC, waist circumference; SBP, systolic blood pressure; DBP, diastolic blood pressure; eGFR, estimated glomerular filtration rate; CVD, cardiovascular disease; LE8, life’s essential 8.

Analysis of the LE8 components revealed significant disparities between the hyperuricemia and non-hyperuricemia groups (Table 2). Participants with hyperuricemia scored lower across several components, including diet quality (38.49 vs. 40.16), physical activity (62.88 vs. 68.49), and sleep health (79.46 vs. 81.44), with *p*-values of <0.001 for each. Notably, BMI scores were substantially lower in the hyperuricemia group (44.46 vs. 63.47), reflecting a higher prevalence of obesity (*p* < 0.001). Blood lipids and glucose scores were also lower among those with hyperuricemia (57.20 vs. 66.37 and 75.89 vs. 83.63, respectively), alongside lower blood pressure scores (55.95 vs. 68.68; *p* < 0.001 for all). Despite the significant differences in these components, the nicotine exposure score was slightly higher in individuals with hyperuricemia (71.20 vs. 71.03; *p* < 0.001), indicating increased nicotine exposure among hyperuricemic individuals.

**Table 2:** Comparison of each component of LE8 scores between individuals with hyperuricemia and individuals without hyperuricemia.

LE8 components	Total *n* = 26,885	Non-hyperuricemia *n* = 21,465	Hyperuricemia *n* = 5,420	*p*-value
HEI-2015 diet score	39.82 ± 31.37	40.16 ± 31.49	38.49 ± 30.88	<0.001
Physical activity score	67.36 ± 43.25	68.49 ± 42.79	62.88 ± 44.74	<0.001
Nicotine exposure score	71.06 ± 39.00	71.03 ± 39.43	71.20 ± 37.24	<0.001
Sleep health score	81.04 ± 25.71	81.44 ± 25.50	79.46 ± 26.46	<0.001
Body mass index score	59.64 ± 33.64	63.47 ± 32.75	44.46 ± 32.85	<0.001
Blood lipids score	64.52 ± 30.21	66.37 ± 29.94	57.20 ± 30.19	<0.001
Blood glucose score	82.07 ± 26.43	83.63 ± 25.90	75.89 ± 27.56	<0.001
Blood pressure score	66.12 ± 32.20	68.68 ± 31.79	55.95 ± 31.78	<0.001

LE8, life’s essential 8; HEI, healthy eating index.

### 3.3 Association between LE8 and hyperuricemia

The logistic regression analysis revealed a significant inverse relationship between LE8 scores and the likelihood of hyperuricemia. In Model 1, which was unadjusted, a 10-point increase in the LE8 score corresponded to a 29% reduction in the odds of hyperuricemia (OR: 0.71, 95% CI: 0.69–0.72). This association remained robust in adjusted models: Model 2 (adjusted for age and sex) and Model 3 (further adjusted for race/ethnicity, PIR, education level, drinking, eGFR, and CVD), with ORs of 0.73 (95% CI: 0.71–0.75) and 0.73 (95% CI: 0.72–0.75), respectively (P for trend <0.001 for all models; Table 3).

**Table 3:** Association of the LE8 scores with hyperuricemia.

Exposure	Model 1	Model 2	Model 3
Continuous (per 10 points)	0.71 (0.69, 0.72)	0.73 (0.71, 0.75)	0.73 (0.72, 0.75)
Categorical			
Low (0–49)	Reference	Reference	Reference
Moderate (50–79)	0.55 (0.51, 0.60)	0.58 (0.54, 0.63)	0.63 (0.58, 0.69)
High (80–100)	0.18 (0.16, 0.21)	0.22 (0.20, 0.25)	0.24 (0.21, 0.27)
*P* for trend	<0.001	<0.001	<0.001

Model 1: Non-adjusted. Model 2: Adjusted for age and sex. Model 3: Adjusted for age, sex, race/ethnicity, PIR, educational level, drinking, eGFR, and CVD. LE8, life’s essential 8; PIR, poverty income ratio; eGFR, estimated glomerular filtration rate; CVD, cardiovascular disease.

When examining individual components of the LE8 score, several components showed significant associations with hyperuricemia. Higher scores in diet, physical activity, sleep health, BMI, blood lipids, blood glucose, and blood pressure were each linked to lower odds of hyperuricemia. For instance, a high physical activity score (80–100) was significantly associated with a lower likelihood of hyperuricemia compared to a low score (0–49), with an OR of 0.86 (95% CI: 0.80–0.93) in the fully adjusted model (P for trend <0.0001). Conversely, higher nicotine exposure scores were associated with increased odds of hyperuricemia, with those in the moderate category (50–79) having an OR of 1.32 (95% CI: 1.20–1.45) compared to the reference group (P for trend <0.05; Table 4).

**Table 4:** Associations between components of LE8 scores and hyperuricemia.

Exposure	Model 1	Model 2	Model 3
HEI-2015 diet score			
Continuous (per 10 points)	0.98 (0.97, 0.99)	0.97 (0.96, 0.98)	0.98 (0.97, 0.99)
Categorical			
Low (0–49)	Reference	Reference	Reference
Moderate (50–79)	1.04 (0.97, 1.11)	0.97 (0.91, 1.05)	1.03 (0.96, 1.11)
High (80–100)	0.88 (0.81, 0.94)	0.78 (0.72, 0.84)	0.87 (0.80, 0.94)
P for trend	0.0025	<0.0001	0.0030
Physical activity score			
Continuous (per 10 points)	0.97 (0.96, 0.98)	0.98 (0.97, 0.99)	0.98 (0.98, 0.99)
Categorical			
Low (0–49)	Reference	Reference	Reference
Moderate (50–79)	0.88 (0.76, 1.02)	0.96 (0.83, 1.11)	1.02 (0.88, 1.19)
High (80–100)	0.76 (0.72, 0.81)	0.83 (0.78, 0.88)	0.86 (0.80, 0.93)
*P* for trend	<0.0001	<0.0001	<0.0001
Nicotine exposure score			
Continuous (per 10 points)	1.00 (0.99, 1.01)	1.00 (0.99, 1.01)	1.01 (1.01, 1.02)
Categorical			
Low (0–49)	Reference	Reference	Reference
Moderate (50–79)	1.50 (1.37, 1.63)	1.20 (1.10, 1.32)	1.32 (1.20, 1.45)
High (80–100)	1.01 (0.94, 1.09)	1.02 (0.94, 1.10)	1.15 (1.06, 1.26)
P for trend	0.0300	0.5802	0.0237
Sleep health score			
Continuous (per 10 points)	0.97 (0.96, 0.98)	0.97 (0.96, 0.98)	0.98 (0.97, 1.00)
Categorical			
Low (0–49)	Reference	Reference	Reference
Moderate (50–79)	0.87 (0.80, 0.96)	0.88 (0.80, 0.97)	0.96 (0.87, 1.06)
High (80–100)	0.81 (0.75, 0.88)	0.81 (0.75, 0.88)	0.89 (0.82, 0.96)
P for trend	<0.0001	<0.0001	0.0023
Body mass index score			
Continuous (per 10 points)	0.84 (0.84, 0.85)	0.84 (0.83, 0.84)	0.83 (0.83, 0.84)
Categorical			
Low (0–49)	Reference	Reference	Reference
Moderate (50–79)	0.50 (0.47, 0.54)	0.46 (0.43, 0.50)	0.46 (0.43, 0.50)
High (80–100)	0.24 (0.22, 0.26)	0.25 (0.22, 0.27)	0.24 (0.22, 0.27)
P for trend	<0.0001	<0.0001	<0.0001
Blood lipids score			
Continuous (per 10 points)	0.91 (0.90, 0.91)	0.92 (0.91, 0.93)	0.91 (0.90, 0.92)
Categorical			
Low (0–49)	Reference	Reference	Reference
Moderate (50–79)	0.68 (0.63, 0.74)	0.76 (0.70, 0.82)	0.78 (0.72, 0.84)
High (80–100)	0.57 (0.54, 0.61)	0.61 (0.57, 0.65)	0.56 (0.52, 0.60)
P for trend	<0.0001	<0.0001	<0.0001
Blood glucose score			
Continuous (per 10 points)	0.90 (0.89, 0.91)	0.93 (0.92, 0.94)	0.94 (0.93, 0.95)
Categorical			
Low (0–49)	Reference	Reference	Reference
Moderate (50–79)	0.89 (0.81, 0.98)	0.94 (0.86, 1.03)	1.07 (0.97, 1.18)
High (80–100)	0.48 (0.44, 0.52)	0.60 (0.55, 0.65)	0.65 (0.59, 0.72)
P for trend	<0.0001	<0.0001	<0.0001
Blood pressure score			
Continuous (per 10 points)	0.89 (0.88, 0.90)	0.91 (0.90, 0.92)	0.92 (0.91, 0.93)
Categorical			
Low (0–49)	Reference	Reference	Reference
Moderate (50–79)	0.66 (0.61, 0.71)	0.76 (0.70, 0.82)	0.84 (0.77, 0.91)
High (80–100)	0.46 (0.43, 0.49)	0.59 (0.54, 0.64)	0.62 (0.57, 0.68)
P for trend	<0.0001	<0.0001	<0.0001

Model 1: Non-adjusted. Model 2: Adjusted for age and sex. Model 3: Adjusted for age, sex, race/ethnicity, PIR, educational level, drinking, eGFR, and CVD. LE8, life’s essential 8; HEI, healthy eating index; PIR, poverty income ratio; eGFR, estimated glomerular filtration rate; CVD, cardiovascular disease.

**Figure 2:**
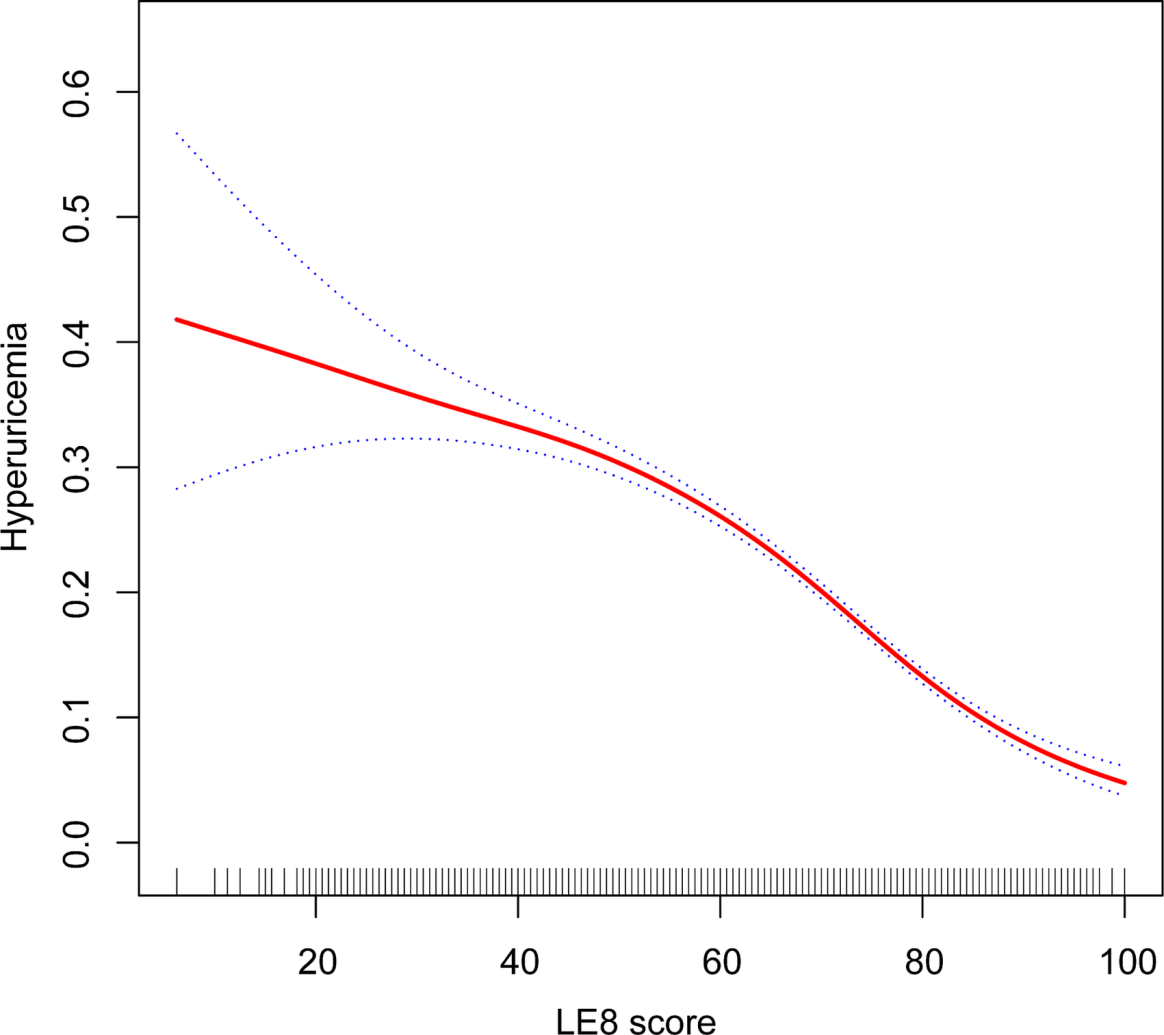
Relationship between LE8 and hyperuricemia. LE8, Life’s Essential 8.

The analysis using a GAM revealed a linear relationship between LE8 scores and the probability of hyperuricemia. Initially, the exploration aimed to identify any nonlinear associations; however, the results indicated a clear linear trend. As the LE8 score increased, the probability of hyperuricemia consistently decreased. This linear association suggests a potential protective impact of higher LE8 scores on reducing the risk of hyperuricemia (Figure 2).

### 3.4 Stratified analysis of the association between LE8 and hyperuricemia

**Figure 3:**
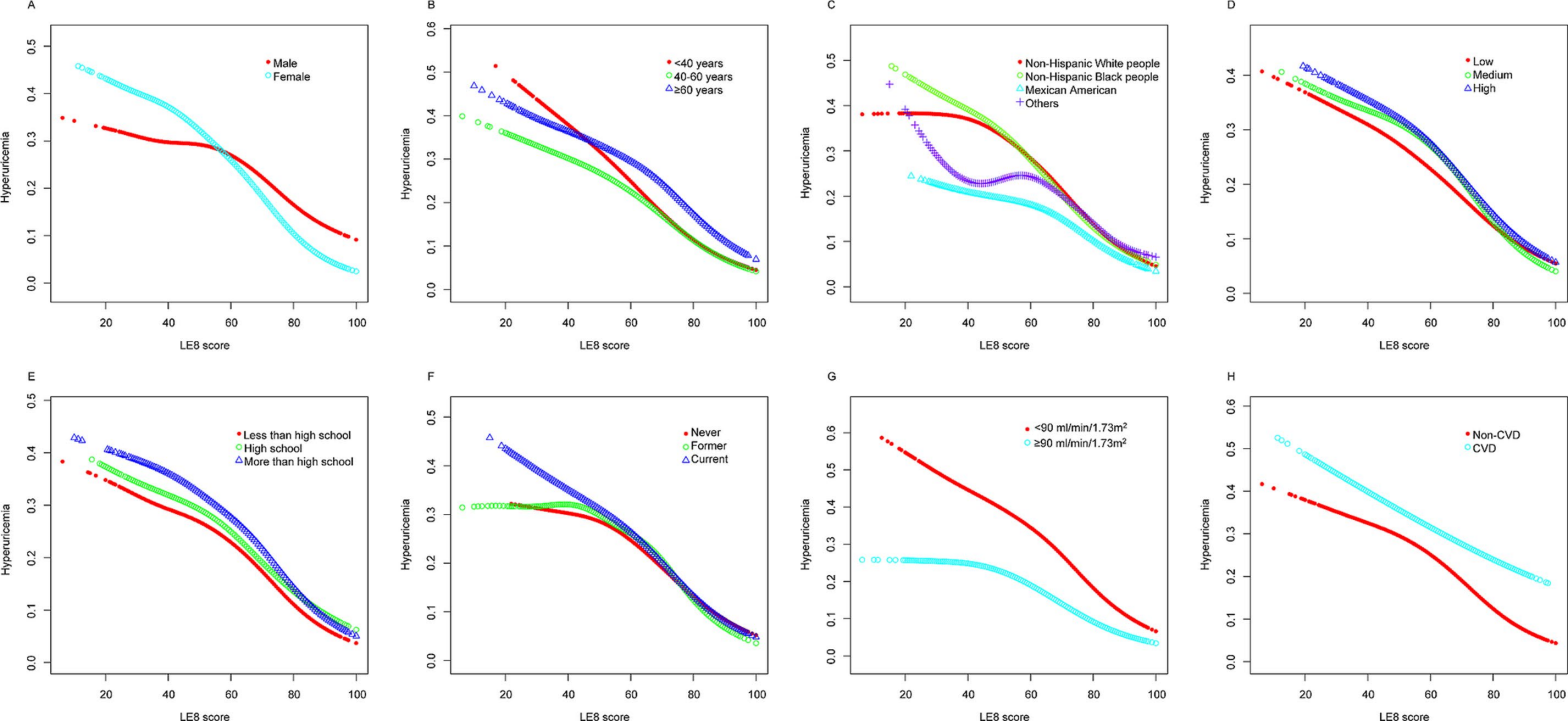
Logistic regression analysis for subgroup interaction. *Each stratification adjusted for all the factors (age, sex, race/ethnicity, PIR, educational level, drinking, eGFR, and CVD) except the stratification factor itself. OR, odds ratio; CI, confidence interval; PIR, poverty income ratio; eGFR, estimated glomerular filtration rate; CVD, cardiovascular disease.

**Figure 4:**
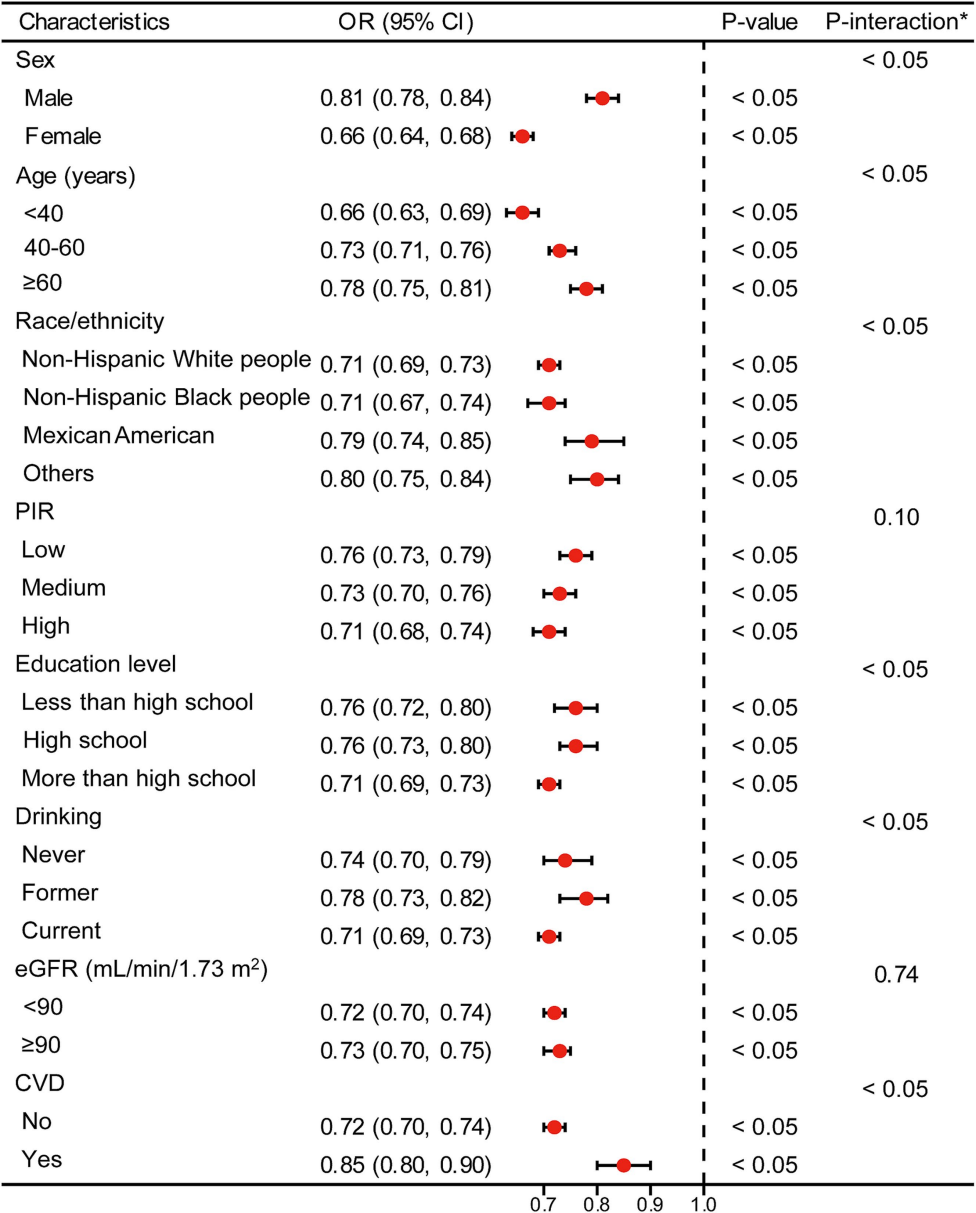
Stratified analyses [by (A) sex; (B) age; (C) race/ethnicity; (D) PIR; (E) education level; (F) drinking; (G) eGFR; (H) CVD] between LE8 and hyperuricemia using GAM. *Each generalized additive model and smooth curve fitting was adjusted for all factors, including age, sex, race/ethnicity, PIR, educational level, drinking, eGFR, and CVD, except for the stratification factor itself. PIR, poverty income ratio; eGFR, estimated glomerular filtration rate; CVD, cardiovascular disease; LE8, Life’s Essential 8; GAM, generalized additive model.

Figures 3, 4 present stratified analyses to verify the association between LE8 scores and hyperuricemia across different population subgroups. Figure 3 employs logistic regression to assess interactions within various subgroups, while Figure 4 uses GAMs to further explore these associations, maintaining the same subgroup categories as in Figure 3.

In Figure 3, logistic regression analysis demonstrates that higher LE8 scores are consistently associated with lower odds of hyperuricemia across all subgroups, including sex, age, race/ethnicity, PIR, education level, drinking status, eGFR, and CVD status. For example, males and females both show a significant reduction in the odds of hyperuricemia with higher LE8 scores, with OR of 0.81 (95% CI: 0.78–0.84) and 0.66 (95% CI: 0.64–0.68), respectively (*p* < 0.05 for both). This pattern holds across other subgroups, indicating that the beneficial effects of higher LE8 scores on reducing hyperuricemia risk are robust and broadly applicable.

Figure 4 corroborates these findings using GAMs, revealing a linear decrease in the probability of hyperuricemia with increasing LE8 scores across the same subgroups. The analyses highlight that irrespective of demographic or health-related factors, higher LE8 scores consistently correlate with lower hyperuricemia risk.

## 4 Discussion

This study explored the relationship between LE8 scores and hyperuricemia among adults in the United States using data from the NHANES 2005–2018. The results revealed that higher LE8 scores are significantly associated with lower odds of hyperuricemia. Specifically, for every 10-point increase in the LE8 score, the likelihood of hyperuricemia decreased by 27%. This inverse relationship remained consistent across various subgroups, including differences in sex, age, race/ethnicity, PIR, educational level, drinking status, eGFR, and CVD status. These findings suggest that higher LE8 scores, which reflect better cardiovascular health, may lower the risk of hyperuricemia by improving metabolic status.

LE8, as an enhanced framework for assessing and promoting cardiovascular health, encompasses a comprehensive range of behavioral, psychological, and social factors (6). It has been widely demonstrated to be closely associated with cardiovascular health and mortality risk (21, 22). Concurrently, numerous studies have shown that LE8 is closely related to metabolic diseases such as diabetes mellitus, hypertension, and metabolic syndrome (MetS). Pang et al. found that higher LE8 scores were associated with lower insulin resistance (IR) ratios (OR per 10-unit increase, 0.57 [95% CI, 0.54–0.61]) in a fully adjusted model (23). Ueno et al. demonstrated that the four health behavior factors involved in LE8 can stratify the risk of diabetes and hypertension, suggesting that improving these health behaviors can help prevent these conditions in the general population (24). Tian et al.’s study also identified a negative correlation between LE8 and hypertension risk (25). Similarly, Gou et al. found that elevated LE8 scores were linked to a decreased incidence of MetS. Specifically, the high LE8 subgroup experienced a 79.73% reduction in MetS risk compared to the low LE8 subgroup (OR = 0.20 [95% CI, 0.09–0.47]) (26). Additionally, both Cao et al. and Yang et al. found a negative association between LE8 and metabolic health and the risk of cardiometabolic diseases (27, 28). Therefore, the relationship between LE8 and hyperuricemia, which is closely related to metabolic factors such as blood pressure, blood lipid, and blood glucose, warrants further study and discussion.

Recent studies have explored the association between LE8 and hyperuricemia, yet certain limitations in methodology and scope highlight the need for further investigation. Yang et al. conducted an analysis of the Kailuan cohort, revealing that higher LE8 scores were associated with a lower risk of hyperuricemia (12). However, the predominantly male, middle-aged population in this study limits the generalizability of its findings to more diverse groups (29). Similarly, Wang et al. examined LE8 in a multi-ethnic Chinese cohort (CMEC), showing an inverse association with hyperuricemia, but the study was geographically restricted to China, leaving the results less applicable to non-Asian populations (11, 30). Wang and Meng extended this research by using NHANES data from 2009 to 2020 to assess the relationship between LE8 and hyperuricemia in U.S. adults (13). While their study offered valuable insights, it combined data from the 2019–2020 cycle with the 2017–2018 cycle, which may introduce methodological concerns, as the NHANES protocol combines these cycles (31). This potential duplication in the dataset could affect the robustness of their findings. Additionally, although they adjusted for some confounders, key lifestyle factors such as alcohol consumption were not fully considered, potentially impacting the accuracy of their risk estimates (32). Han et al. also investigated the association between LE8 and hyperuricemia in a U.S. cohort, utilizing restricted cubic spline models to analyze non-linear relationships (14). However, their study focused on overall LE8 scores and long-term health outcomes, such as all-cause mortality, without fully addressing the contribution of individual LE8 components, such as BMI, blood lipids, and blood glucose, to hyperuricemia risk.

Our findings are in alignment with previous research, highlighting the importance of individual components of LE8 in their association with serum uric acid levels and hyperuricemia. For instance, Fang et al. observed that patients with dyslipidemia were 1.88 times more likely to develop hyperuricemia compared to those without dyslipidemia (95% CI: 1.84–1.92) (33). Similarly, Nie et al. demonstrated that adherence to a healthy diet, as measured by the HEI-2015, significantly lowers serum uric acid levels and decreases the risk of hyperuricemia (34). Additionally, Hong et al. reported that increased physical activity correlates with reduced serum uric acid levels and a lower incidence of hyperuricemia (35). These studies support our findings, indicating that the cumulative effect of these health behaviors, as reflected by LE8 scores, offers substantial protection against hyperuricemia.

Our study also found that higher nicotine exposure scores were associated with increased odds of hyperuricemia, a finding that contrasts with some prior studies. While Wang and Krishnan reported that cigarette smoking is associated with a lower risk of incident gout, possibly due to the inactivation of xanthine oxidase by the cyanides in cigarettes (36), other studies suggest a more complex relationship. For instance, Jee et al. indicated that the association between smoking and gout risk remains controversial (37), with some studies showing decreased risk and others showing no significant association. Additionally, Gee Teng et al. highlighted the potential risk of hyperuricemia in smokers, attributing it to the oxidative stress and its impact on uric acid metabolism (38). These mixed findings suggest that the relationship between nicotine exposure and hyperuricemia may be influenced by various factors, including smoking intensity, duration, and concurrent health behaviors (36–39). Therefore, while some studies indicate that smoking may offer limited protection against hyperuricemia, this should not be interpreted as an endorsement of smoking, given its well-documented health risks.

In addition, some studies have reported weak or inconsistent associations between other individual lifestyle factors, such as sleep, and serum uric acid levels. For example, a cross-sectional study involving Chinese government employees examined the relationship between nighttime sleep duration and hyperuricemia risk. The findings indicated that participants sleeping less than 7 h per night had a higher risk of hyperuricemia compared to those sleeping 7–8 h (40). Conversely, other research suggested that poor sleep quality might be associated with lower serum uric acid levels, particularly in patients with Parkinson’s disease (41). Additionally, some studies found no significant association between blood pressure and serum uric acid levels (42). Although many epidemiological studies have identified a strong correlation between hyperuricemia and hypertension, some evidence suggests that lowering uric acid levels does not substantially improve blood pressure control in hypertensive patients (43). Furthermore, variations in uric acid levels may interact with other metabolic factors, such as insulin resistance or dyslipidemia, contributing to the development of hypertension (44, 45). Therefore, caution is warranted when interpreting these findings, particularly given the complex interplay between uric acid and hypertension (46). Inconsistencies across studies may arise from differences in population characteristics, measurement tools, or definitions of lifestyle factors. For example, studies focusing solely on individual components of LE8 may overlook the cumulative effects of multiple behaviors, which are better captured through a composite LE8 score in our research (6). Additionally, environmental, genetic, and cultural factors across populations may influence how lifestyle affects hyperuricemia risk, highlighting the importance of validating these findings in diverse demographic contexts.

The components of LE8 play a crucial role in mitigating hyperuricemia by addressing its underlying risk factors and mechanisms. Uric acid, a metabolic byproduct of purine catabolism, frequently exhibits elevated levels in individuals with CVD due to shared risk factors, including hypertension, obesity, and insulin resistance (47, 48). By enhancing cardiovascular health, LE8 helps reduce uric acid levels by attenuating systemic inflammation, improving insulin sensitivity, and optimizing renal function, thereby promoting increased uric acid excretion. A diet rich in fiber and antioxidants helps reduce oxidative stress and inflammation, both key contributors to purine accumulation and hyperuricemia development (49, 50). Regular physical activity enhances metabolic health, aids in weight management, and improves insulin sensitivity, thereby facilitating more efficient renal clearance of uric acid (51). Furthermore, avoiding nicotine exposure diminishes systemic inflammation and supports renal function, contributing to better uric acid regulation (52). In contrast, inadequate sleep exacerbates metabolic dysfunction, oxidative stress, and chronic inflammation, thus elevating the risk of hyperuricemia (53). Therefore, improving sleep quality and duration is critical for the prevention and management of hyperuricemia. Recent research suggests that elevated plasma aldosterone concentrations (PAC) in individuals with hypertension significantly contribute to the onset of hyperuricemia and gout by impairing renal excretion of uric acid (54). Managing hypertension and optimizing blood pressure—key aspects of LE8—can help regulate aldosterone levels, mitigating its negative impact on uric acid metabolism. Additionally, dyslipidemia and hyperglycemia exacerbate metabolic dysfunction, resulting in increased uric acid production and reduced excretion (55). Effective management of lipid levels and blood glucose, both integral to LE8, supports metabolic health and reduces the risk of hyperuricemia. Finally, a higher BMI is associated with elevated uric acid levels due to overproduction and impaired excretion (56). Maintaining a healthy BMI, another crucial element of LE8, lowers the risk of hyperuricemia.

While our study provides valuable insights into the relationship between LE8 and hyperuricemia, several limitations should be acknowledged. First, the cross-sectional nature of NHANES data limits our ability to infer causality between LE8 scores and hyperuricemia. Longitudinal studies are needed to establish temporal relationships and causality. Second, this study relies on self-reported data for lifestyle factors, such as diet and physical activity, which introduces the possibility of recall bias. However, this limitation is inherent in the design of the NHANES survey. Despite this, NHANES follows standardized procedures to enhance data accuracy, such as using validated questionnaires and trained interviewers. Third, one potential limitation is the presence of unmeasured confounding variables that could influence the observed relationship between LE8 scores and hyperuricemia. For instance, diet and physical activity are closely interrelated and may jointly affect serum uric acid levels. It is possible that diet acts as a mediator between physical activity and hyperuricemia, with healthier dietary habits amplifying the protective effect of physical activity. Future research could explore these potential interactions using mediation models to better understand how individual components of LE8 interact to influence hyperuricemia risk. Additionally, while we adjusted for major covariates such as age, sex, race/ethnicity, and eGFR, residual confounding by other unmeasured factors cannot be entirely ruled out. Fourth, although MICE was used to handle missing data, the method assumes that data were missing at random, which may not always be the case. Finally, although NHANES data are representative of the U.S. population, our findings may not directly apply to populations with different genetic, environmental, or cultural backgrounds.

## 5 Conclusion

This study underscores the importance of comprehensive cardiovascular health, as captured by LE8, in reducing hyperuricemia risk among adults. The LE8 framework, which encompasses diet, physical activity, nicotine exposure, sleep health, BMI, blood lipids, blood glucose, and blood pressure, offers a holistic approach to health that effectively mitigates metabolic disorders such as hyperuricemia. The consistent association between higher LE8 scores and lower hyperuricemia risk indicates a clear metabolic association. This underscores the importance of considering various metabolic determinants in the context of hyperuricemia and suggests potential public health implications for promoting cardiovascular health behaviors.

For future research, longitudinal studies are needed to confirm causal relationships and explore the long-term effects of LE8 adherence across diverse groups. Mediation analysis can further clarify how LE8 components interact to influence uric acid levels. Incorporating objective measures, such as wearable devices for activity tracking and dietary biomarkers, will improve data accuracy and reduce self-reporting bias.

From a public health perspective, promoting LE8 adherence in high-risk groups—such as those with obesity or hypertension—is essential. Community programs, educational campaigns, and policy efforts that improve access to healthy foods and physical activity can help drive sustainable lifestyle changes. Interventional studies in diverse populations will further guide effective prevention strategies for hyperuricemia.

## Data Availability

The raw data supporting the conclusions of this article will be made available by the authors, without undue reservation.

## Footnotes

^1^http://www.EmpowerStats.com
