# Supplementary material for: Association between life’s essential 8 and hyperuricemia among adults in the United States: insights from NHANES 2005–2018
Source: Front Med (Lausanne). 2024 Nov 6;11:1455164. doi: 10.3389/fmed.2024.1455164 (PMC11576210; doi:10.3389/fmed.2024.1455164)
Supplement: Supplementary file 1 [file Data_Sheet_1.DOCX]

**Table S1. Definition and Scoring Approach for the American Heart Association’s LE8 Score**

| **Each component of LE8 scores** | **Quantification and Scoring** |
| --- | --- |
| **HEI-2015 diet score** | For diet, the Healthy Eating Index-2015 diet score percentile is used. The scoring is based on the adherence to a DASH-style diet, with points assigned as follows: 100 points for the 95th percentile or higher (indicating a top or ideal diet), 80 points for the 75th to 94th percentile, 50 points for the 50th to 74th percentile, 25 points for the 25th to 49th percentile, and 0 points for the 1st to 24th percentile (indicating the bottom or least ideal quartile) |
| **Physical activity score** | Physical activity is measured in self-reported minutes of moderate or vigorous physical activity per week. Scoring is as follows: 100 points for 150 minutes or more, 90 points for 120 to 149 minutes, 80 points for 90 to 119 minutes, 60 points for 60 to 89 minutes, 40 points for 30 to 59 minutes, 20 points for 1 to 29 minutes, and 0 points for 0 minutes |
| **Nicotine exposure score** | Nicotine exposure is assessed based on self-reported use of cigarettes or inhaled nicotine-delivery systems. Scoring is as follows: 100 points for never smokers, 75 points for former smokers who quit 5 or more years ago, 50 points for former smokers who quit 1 to less than 5 years ago, 25 points for former smokers who quit less than 1 year ago or currently use inhaled nicotine-delivery systems, and 0 points for current smokers. Additionally, subtract 20 points (unless the score is 0) for living with an active indoor smoker |
| **Sleep health score** | Sleep health is based on self-reported average hours of sleep per night. Scoring is as follows: 100 points for 7 to less than 9 hours, 90 points for 9 to less than 10 hours, 70 points for 6 to less than 7 hours, 40 points for 5 to less than 6 or 10 or more hours, 20 points for 4 to less than 5 hours, and 0 points for less than 4 hours |
| **Body mass index score** | Body mass index is calculated by dividing body weight in kilograms by height in meters squared. Scoring is as follows: 100 points for a BMI of less than 25, 70 points for a BMI of 25.0 to 29.9, 30 points for a BMI of 30.0 to 34.9, 15 points for a BMI of 35.0 to 39.9, and 0 points for a BMI of 40.0 or more |
| **Blood lipids score** | Blood lipids are measured by plasma total and HDL-cholesterol with the calculation of non-HDL-cholesterol. Scoring is as follows: 100 points for non-HDL-cholesterol less than 130 mg/dL, 60 points for 130 to 159 mg/dL, 40 points for 160 to 189 mg/dL, 20 points for 190 to 219 mg/dL, and 0 points for 220 mg/dL or more. If the level is drug-treated, subtract 20 points |
| **Blood glucose score** | Blood glucose is measured by fasting blood glucose or casual hemoglobin A1c. Scoring is as follows: 100 points for no history of diabetes and fasting blood glucose less than 100 mg/dL (or hemoglobin A1c less than 5.7%), 60 points for no diabetes and fasting blood glucose 100 to 125 mg/dL (or hemoglobin A1c 5.7% to 6.4%) (prediabetes), 40 points for diabetes with hemoglobin A1c less than 7.0%, 30 points for diabetes with hemoglobin A1c 7.0% to 7.9%, 20 points for diabetes with hemoglobin A1c 8.0% to 8.9%, 10 points for diabetes with hemoglobin A1c 9.0% to 9.9%, and 0 points for diabetes with hemoglobin A1c 10.0% or more |
| **Blood pressure score** | Blood pressure is measured by appropriately taken systolic and diastolic blood pressure. Scoring is as follows: 100 points for less than 120/80 mmHg (optimal), 75 points for 120-129/<80 mm Hg (elevated), 50 points for 130-139 or 80-89 mmHg (stage I hypertension), 25 points for 140-159 or 90-99 mmHg, and 0 points for 160 or more or 100 or more mmHg. Subtract 20 points if the level is treated |

**References:**

- Lloyd-Jones DM, Allen NB, Anderson CAM, et al. Life's Essential 8: Updating and Enhancing the American Heart Association's Construct of Cardiovascular Health: A Presidential Advisory From the American Heart Association. Circulation. Aug 2, 2022;146(5)

**Table S2. Healthy eating index-2015 components & scoring standards**

| **Component** | **Maximum Points** | **Standard for Maximum Score** | **Standard for Minimum Score of Zero** |
| --- | --- | --- | --- |
| **Adequacy** |  |  |  |
| Total Fruits | 5 | ≥0.8 cup equiv. per 1000 kcal | No Fruit |
| Whole Fruits | 5 | ≥0.4 cup equiv. per 1000 kcal | No Whole Fruit |
| Total Vegetables | 5 | ≥1.1 cup equiv. per 1000 kcal | No Vegetables |
| Greens and Beans | 5 | ≥0.2 cup equiv. per 1000 kcal | No Dark Green Vegetables or Legumes |
| Whole Grains | 10 | ≥1.5 oz equiv. per 1000 kcal | No Whole Grains |
| Dairy | 10 | ≥1.3 cup equiv. per 1000 kcal | No Dairy |
| Total Protein Foods | 5 | ≥2.5 oz equiv. per 1000 kcal | No Protein Foods |
| Seafood and Plant Proteins | 5 | ≥0.8 oz equiv. per 1000 kcal | No Seafood or Plant Proteins |
| Fatty Acids | 10 | (PUFAs + MUFAs)/SFAs ≥2.5 | (PUFAs + MUFAs)/SFAs ≤1.2 |
| **Moderation** |  |  |  |
| Refined Grains | 10 | ≤1.8 oz equiv. per 1000 kcal | ≥4.3 oz equiv. per 1000 kcal |
| Sodium | 10 | ≤1.1 gram per 1000 kcal | ≥2.0 grams per 1000 kcal |
| Added Sugars | 10 | ≤6.5% of energy | ≥26% of energy |
| Saturated Fats | 10 | ≤8% of energy | ≥16% of energy |

**Notes:**

- Intakes between the minimum and maximum standards are scored proportionately.
- Total Fruits includes 100% fruit juice.
- Whole Fruits includes all forms except juice.
- Total Vegetables includes legumes (beans and peas).
- Greens and Beans includes legumes (beans and peas).
- Dairy includes all milk products such as fluid milk, yogurt, and cheese, and fortified soy beverages.
- Total Protein Foods includes legumes (beans and peas).
- Seafood and Plant Proteins includes seafood, nuts, seeds, soy products (other than beverages), and legumes (beans and peas).
- Fatty Acids ratio of poly- and monounsaturated fatty acids (PUFAs and MUFAs) to saturated fatty acids (SFAs).

**References:**

- Krebs-Smith SM, Pannucci TE, Subar AF, et al. Update of the Healthy Eating Index: HEI-2015. J Acad Nutr Diet. Sep 2018;118(9):1591-1602.
